# Biophysical Analysis of Apolipoprotein E3 Variants Linked with Development of Type III Hyperlipoproteinemia

**DOI:** 10.1371/journal.pone.0027037

**Published:** 2011-11-01

**Authors:** Dimitra Georgiadou, Angeliki Chroni, Alexander Vezeridis, Vassilis I. Zannis, Efstratios Stratikos

**Affiliations:** 1 Protein Chemistry Laboratory, National Centre for Scientific Research Demokritos, Agia Paraskevi, Athens, Greece; 2 Institute of Biology, National Centre for Scientific Research Demokritos, Agia Paraskevi, Athens, Greece; 3 Molecular Genetics, Departments of Medicine and Biochemistry, Whitaker Cardiovascular Institute, Boston University School of Medicine, Boston, Massachusetts, United States of America; University of Queensland, Australia

## Abstract

**Background:**

Apolipoprotein E (apoE) is a major protein of the lipoprotein transport system that plays important roles in lipid homeostasis and protection from atherosclerosis. ApoE is characterized by structural plasticity and thermodynamic instability and can undergo significant structural rearrangements as part of its biological function. Mutations in the 136–150 region of the N-terminal domain of apoE, reduce its low density lipoprotein (LDL) receptor binding capacity and have been linked with lipoprotein disorders, such as type III hyperlipoproteinemia (HLP) in humans. However, the LDL-receptor binding defects for these apoE variants do not correlate well with the severity of dyslipidemia, indicating that these variants may carry additional properties that contribute to their pathogenic potential.

**Methodology/Principal Findings:**

In this study we examined whether three type III HLP predisposing apoE3 variants, namely R136S, R145C and K146E affect the biophysical properties of the protein. Circular dichroism (CD) spectroscopy revealed that these mutations do not significantly alter the secondary structure of the protein. Thermal and chemical unfolding analysis revealed small thermodynamic alterations in each variant compared to wild-type apoE3, as well as effects in the reversibility of the unfolding transition. All variants were able to remodel multillamelar 1,2-Dimyristoyl-*sn*-glycero-3-phosphocholine (DMPC) vesicles, but R136S and R145C had reduced kinetics. Dynamic light scattering analysis indicated that the variant R136S exists in a higher-order oligomerization state in solution. Finally, 1-anilinonaphthalene-8-sulfonic acid (ANS) binding suggested that the variant R145C exposes a larger amount of hydrophobic surface to the solvent.

**Conclusions/Significance:**

Overall, our findings suggest that single amino acid changes in the functionally important region 136–150 of apoE3 can affect the molecule's stability and conformation in solution and may underlie functional consequences. However, the magnitude and the non-concerted nature of these changes, make it unlikely that they constitute a distinct unifying mechanism leading to type III HLP pathogenesis.

## Introduction

Apolipoprotein E (apoE) is a major protein of the lipoprotein transport system that plays critical role in the protection from atherosclerosis and dyslipidemia [1]. ApoE mediates the hepatic clearance of lipoprotein remnants from circulation serving as a ligand for the low-density lipoprotein (LDL) receptor [2]. Additionally, apoE interacts with other members of the LDL receptor family and cell-surface heparan sulfate proteoglycans (HSPGs)[3], [4]. ApoE is also involved in cholesterol efflux processes [5], [6] and thus may contribute to cell and tissue cholesterol homeostasis [7], [8]. ApoE contains 299 residues and has three common isoforms (apoE2, apoE3, apoE4) in the general population, each differing in the amino acid positions 112 and 158 [9], [10]. ApoE3, the most common form, contains cysteine at position 112 and arginine at position 158, whereas apoE2 has two cysteine residues and apoE4 has two arginine residues at these two positions. ApoE4 is a major genetic risk factor for late-onset Alzheimer's disease [11], [12].

In the lipid-free state apoE is folded into two independent structural domains. Digestion with thrombin produces a 22-kDa N-terminal fragment (residues 1 to 191) and a 10-kDa C-terminal fragment (residues 216 to 299) [13], [14]. X-ray crystallography studies showed that the N-terminal domain is folded into a four-helix bundle of amphipathic α-helices spanning residues 24-164 that are stabilized by hydrophobic interactions and salt bridges [15], [16], [17]. The C-terminal domain is highly α-helical, as determined by computer modeling and circular dichroism spectroscopy [13], [18], [19], but its exact structure is unknown. In apoE4, the N- and C-terminal domains interact differently than in the other isoforms, since Arg-112 results in the orientation of the Arg-61 side chain in the N-terminal domain of apoE4 away from the four-helix bundle. This orientation allows Arg-61 to form a salt bridge with Glu-255 in the C-terminal domain [16], [20]. In apoE3 and apoE2 Arg-61 and Glu-255 do not interact.

Homozygosity for the apoE2 isoform, which has defective *in vitro* binding capacity to the LDL receptor, is present in the vast majority of subjects with type III hyperlipoproteinemia (HLP) [21], [22]. This lipid disorder is characterized by xanthomas, elevated plasma cholesterol and triglyceride levels, and is associated with premature atherosclerosis in humans and experimental animal models of the disease [23], [24], [25]. Besides apoE2, which is associated with a recessive form of type III HLP, a variety of rare naturally-occurring apoE mutations have also been described that are associated with a dominant mode of inheritance of type III HLP which is expressed at an early age [22]. Most of these apoE mutations are between residues 136 to 150 [22], [26]. Specifically, heterozygosity for the apoE variant apoE3[R136S] (previously designated apoE2 Christchurch) with the apoE2 allele results in type III HLP [27]. *In vitro* studies showed that lipid-bound apoE3[R136S] displays a reduction of 60% in LDL receptor binding capacity as compared to wild-type apoE3 [28]. Additionally, it has been shown that homozygosity for apoE3[R136S] and heterozygocity for apoE3[R136S] with all apoE alleles result also in type III HLP, but the penetrance of the disease is incomplete [29]. Homozygosity and heterozygocity for the apoE variant apoE3[R145C] has also been associated with severe type III HLP [30], [31]. Binding studies showed that lipid-bound apoE3[R145C] has 55% reduced LDL receptor binding capacity compared to apoE3 and the 22-kDa domain of lipid-bound apoE3[R145C] has 48% reduced HSPG binding capacity compared to apoE3-22kDa [30], [32]. Finally, heterozygosity for the apoE variant apoE3[K146E] with the apoE3 allele resulted in a dominant expression of type III HLP [33], [34], [35]. Lipid-bound apoE3[K146E] had less than 10% binding capacity to the LDL receptor and HSPG compared with apoE3 [34], [35], [36].

Interestingly, receptor binding defects for these apoE variants do not correlate well with the severity of dyslipidemia, which can be influenced by other secondary factors [28], [37], [38]. Furthermore, the degree of penetrance varies depending on the mutation [22]. Factors may affect the severity of dyslipidemia in patients carrying these mutations include the natural apoE polymorphism which affects the distribution of apoE to different lipoprotein classes [39], interaction of apoE with HSPGs [40] or additional genetic, hormonal, or environmental factors, such as obesity, hypothyroidism, estrogen status, or diabetes [41].

**Figure 1:**
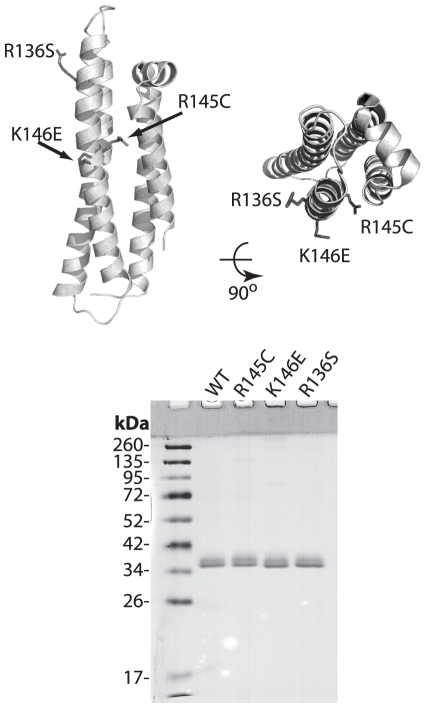
*Top,* Cartoon representation of the N-terminal structure of human apoE3 (PDB code: 1LPE) indicating the location of the mutations; protein is depicted in side and top view. *Bottom*, SDS-PAGE analysis of the recombinant apoE3 variants and wild-type protein. Molecular mass marker bands are indicated. Images were prepared using PyMol 1.3 (www.pymol.org).

Several studies have suggested that the structural and biophysical properties of apoE can dictate the function of the protein and have provided insight into the mechanisms by which apoE is involved in cardiovascular and neurological diseases [42], [43], [44], [45], [46], [47], [48], [49]. In the current study we tested whether three naturally-occurring point variants of apoE3 linked with the development of type III HLP affect the thermodynamic stability of the molecule. The R145C, K146E and R136S mutations are all located within helix 4 of the N-terminal four-helix bundle of the protein (Figure 1) and encode for non-conservative amino acid substitutions. We therefore hypothesized that such drastic residue changes may affect protein stability and conformation. Furthermore, we hypothesized that shared changes in the biophysical properties of these variants may support disease phenotypes. To test this hypothesis, we employed circular dichroism (CD) spectrometry to compare the secondary structure and thermal denaturation profiles of the variants compared to the wild-type apoE3. We used fluorescence spectroscopy to analyze their chemical denaturation profiles and 1-anilinonaphthalene-8-sulfonic acid (ANS) hydrophobic probe binding to quantitate solvent-exposed hydrophobic surfaces. Furthermore, we used dynamic light scattering to assess the solution oligomerization state of the apoE3 variants. Finally, we measured the kinetics of remodeling of multilamellar 1,2-Dimyristoyl-*sn*-glycero-3-phosphocholine (DMPC) vesicles by each variant. Overall, our data point to changes in some of the biophysical parameters of each variant compared to the wild-type form suggesting that even single-amino acid changes in this region of the protein can influence the stability and conformation of apoE3. These changes however are not concerted amongst the three mutations, suggesting that the alterations in the biophysical properties of these apoE3 variants do not constitute a single unifying mechanism underlying their pathogenic potential.

## Materials and Methods

### Site-directed mutagenesis and adenovirus construction

ApoE3 variants were constructed using the QuikChange-XL (Stratagene) side-directed mutagenesis kit, using as a template the vector pGEM7-apoE3 containing exons II, III and IV of human apoE3 gene [50]. To generate the variants R145C, K146E and R136S the following sets of primers were used: R136S-f (5′-GAG GAG CTG CGG GTG **A**GC CTC GCC TCC CAC CTG-3′) and R136S-r (5′-CAG GTG GGA GGC GAG GC**T** CAC CCG CAG CTC CTC-3′) for the first variant, R145C-f (5′- CAC CTG CGC AAG CTG **T**GT AAG CGG CTC CTC CGC-3′) and R145-r (5′-GCG GAG GAG CCG CTT AC**A** CAG CTT GCG CAG GTG-3′) for the second variant, K146E-f (5′-CTG CGC AAG CTG CGT **G**AG CGG CTC CTC CGC GAT-3′) and K146E-r (5′-ATC GCG GAG GAG CCG CT**C** ACG CAG CTT GCG CAG-3′) for the third variant. Successful mutagenesis was confirmed by sequencing the template vector. Recombinant adenoviruses were constructed as previously described [50] using the Ad-Easy-1 system [51]. Correct clones were propagated in RecA DH5α cells [51]. The recombinant adenoviral vectors were linearized with PacI and used to infect 911 cells [52]. Human embryonic kidney 293 cell cultures (ATCC number CRL-1573) were used for large-scale amplification of the viruses [51].

### Protein expression

Human astrocytoma HTB13 cells (ATCC number HTB-13, designation SW 1783) grown in roller bottles were infected with the appropriate recombinant adenoviruses with a multiplicity of infection of 20 pfu/cell. The medium (Leibovitz L-15 containing 1% v/v penicillin and streptomycin and 2% v/v heat-inactivated horse serum) was discarded 24 hours post-infection and the cells in the roller bottles were rinsed with Dulbecco's phosphate-buffered saline (DPBS). The DPBS was discarded and Leibovitz L-15 medium containing 1% v/v penicillin and streptomycin was added. The medium was harvested 24 hours later and the harvest was repeated at 24-hour intervals for 4-6 days. The collected media were centrifuged and filtered through 0.2 µm sterile filters and were stored at -80°C. The amount of the expressed wild-type and variant apoE3 forms was estimated by SDS-PAGE. The medium containing the recombinant protein was lyophilized to reduce total volume to ∼200 ml and then extensively dialyzed against 25 mM of ammonium bicarbonate. The dialyzed medium was lyophilized and the dried recombinant protein stored at −80°C.

### Protein purification

Recombinant wild-type and variant apoE3 forms were purified by a lipidation/de-lipidation protocol involving formation of proteoliposomes, density gradient ultracentrifugation of proteoliposomes to isolate pure apoE3 and then delipidation of purified apoE3 followed by chemical refolding. The procedure is described bellow for delipidating one mg of recombinant apoE3:

9.5 mg of β-oleoyl-γ-palmitoyl-L-α-phosphatidylcholine (POPC, Avanti Polar Lipids) and 0.47 mg of cholesterol (Sigma) / mg of apoE3 to be delipidated were mixed and dissolved in ∼10 ml of chloroform:methanol solution (2∶1 v/v). The solution was dried under gas nitrogen. A volume of 0.42 ml of salt buffer (10 mM Tris-HCl, 150 mM NaCl, 0.01% EDTA, pH8.0) per mg of apoE3 was added in the flask with the dried cholesterol and phospholipids. The flask was flashed with nitrogen gas, tapped and stirred vigorously for 1 h, at 4°C. Lyophilized recombinant apoE3 was dissolved in salt buffer at 2 mg/ml and was added to the lipid emulsion. The solution was further stirred vigorously for 1 h, at 4°C. Finally, 150 µl/mg apoE3 of a 30 ml/ml sodium cholate stock was added to the solution under stirring, and the mixture was stirred at 4°C for one more hour. The solution of proteoliposomes was then dialyzed 3 times against 10 mM Tris-HCl, 150 mM NaCl, 0.01% EDTA, pH 8.0 at 4 °C.

The proteoliposomes' solution was adjusted to a density of 1.21 g/ml by the addition of KBr. A volume of 13.2 ml of the proteoliposome solution (d = 1.21 g/ml) was transferred to the bottom of 40 ml Beckman tubes and overlaid by 6.6 ml of KBr solution of d = 1.063 g/ml, followed by 6.6 ml of KBr of d = 1.019 g/ml and 13.2 ml of DPBS to the top of the tube. The tubes were transferred to a SW28 rotor and centrifuged at 28000 rpm for 22 hours at 4°C. Following centrifugation, 2 ml fractions were collected from the top. The fractions were dialyzed extensively against ddH_2_O. An aliquot of 20 µl of each fraction were analyzed by SDS-PAGE. The fractions that contained pure apoE3 were pooled and stored at 4°C.

To delipidate the apoE3 containing fractions, aliquots of 3 ml were transferred in sterile siliconized 30 ml Corex™ tubes and then diluted by the addition of 3 ml ammonium bicarbonate buffer (50 mM final concentration). A volume of 23.1 ml of fresh chloroform:methanol (2:1 v/v) solution was added to each tube. The solution was vortexed vigorously, incubated on ice for at least 3 h and the tubes were centrifuged at 8000 rpm using a Beckman JA-20 rotor at 4°C for 30 min to separate the two phases. During this procedure an amorphous precipitate formed between the two phases corresponding to precipitated apoE. The two phases as well as the intermediate precipitate were carefully separated. The top aqueous phase containing apoE was dried under nitrogen gas to remove any residual chloroform and methanol. The bottom organic phase was submitted to the same extraction procedure at least three more times until no more phosphorus could be detected in the aqueous phase as determined by the Bartlett method [53]. The pooled inter-phase precipitate contained pure delipidated apoE3 that had precipitated during the extraction procedure and was refolded as described below. Purified proteins were dried completely and stored in lyophilized form at −80°C.

Before all analyses, lyophilized stocks of wild-type or apoE3 variants were subjected to a refolding protocol as previously described [43]. Briefly the protein powder was dissolved at a final concentration of 0.2 mg/ml in 6 M guanidine hydrochloride in DPBS containing 1 mM DTT. The solution was incubated for 1 h at room temperature and then dialyzed 3 times against DPBS pH 7.4. After dialysis samples were centrifuged at 10000 g for 20 min to remove any precipitated protein. The refolded proteins were quantitated by measuring their absorbance at 280 nm using an extinction coefficient of 1.3 mg^−1^ mL cm^−1^. The proteins were kept at low concentrations (∼0.1 mg/ml) on ice to avoid aggregation. All analyses were performed on freshly refolded protein.

### Circular Dichroism Spectroscopy

Far-UV CD spectra were recorded from 190 to 260 nm at 25°C using a Jasco-715 spectropolarimeter. The cuvette chamber was thermostated using a Jasco PTC-348 WI Peltier temperature controller. Protein samples were at 0.1 mg/ml concentration in DPBS (pH 7.4) containing 1 mM DTT (Applichem). A quartz cuvette with an optical path length of 1 mm was used. Spectra were acquired at 1 nm bandwidth, 8 sec response time, 0.2 nm step size and 50 nm/min scan speed. Each spectrum was calculated as the average of 5 accumulations. The results were corrected by subtracting the buffer baseline.

Helical content was calculated using the molecular ellipticity at 208 and 222 nm as described by Greenfield et al. [54] using the equations: 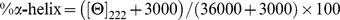



Helical content was also calculated using the DICHROWEB server [55]. The CDSSR method was selected for calculations because it yielded the lowest NRMSD value [56], [57].

For thermal denaturation measurements the change in molar ellipticity at 222 nm was monitored while varying the temperature in the range 20–80°C at a rate of 1°C /min. The thermal denaturation curve was fitted to a Boltzman sigmoidal model curve using the Graphpad Prism™ software. The relative enthalpy change was calculated as described previously [58]. The cooperativity index, *n*, was calculated as previously described [42] (using the Hill equation *n* = (log 81)/log(*T*_0.9_/*T*_0.1_), where *T*_0.9_ and *T*_0.1_ are the temperatures where the unfolding transition has reached a fractional completion of 0.9 and 0.1. For thermal unfolding reversibility measurements, the thermally denatured sample was cooled down to 20°C at a rate of 1°C /min and then re-heated to 80°C while recording the molar ellipticity at 222 nm.

### Chemical denaturation experiments

To record the chemical denaturation profile of apoE3 0.04 mg/mL of freshly refolded protein were added in a 4 mL quartz fluorimeter cuvette in a jacketed holder at 25°C, and the intrinsic tryptophan protein fluorescence was measured at 340 nm after excitation at 295 nm. Small amounts of an 8.0 M guanidine hydrochloride (Applichem) solution in DPBS were gradually added in the cuvette. The contents were mixed by repeated pipetting for 5 sec, incubated in the dark for 2 min and then the fluorescence signal of the sample was measured. The experimental data were fitted to a three-state denaturation model as described by Ekblad et al. [59] using the following equation:
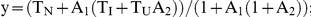



where y is the fluorescence signal, T_N_, T_I,_ T_U_ is the fluorescence signal for the native, intermediate and unfolded state of the protein respectively and A1 and A2 are:
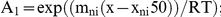


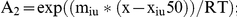



where x is the denaturant concentration, R is the universal gas constant (0.001986 kcal K^−1^ mol^−1^), T is the temperature of the experiment (298 K), x_ni_ and x_iu_ are the mid-transition points for the transition from the native to the intermediate state and from the intermediate to the unfolded state respectively. Finally, m_ni_ and m_iu_ correspond to the slope of the mid-transition points from the native to the intermediate state and from the intermediate to the unfolded state respectively. The relative change in Gibbs-free energy during the chemical denaturation was calculated as follows [59]:
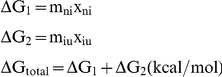



### ANS Fluorescence

1,8 ANS (1-anilinonaphthalene-8-sulfonic acid, Sigma-Aldrich) was dissolved into dimethylsulfoxide (DMSO) to a final concentration of 50 mM (ANS stock solution) and stored at −20°C. Freshly refolded wild-type and apoE3 variants in 1x DPBS pH 7.4, 0.1 mM DTT at 0.04 mg/ml were inserted into a 4 mL quartz fluorimeter cuvette (Hellma, Germany). Fluorescence measurements were preformed in a Quantamaster 4 fluorescence spectrometer (Photon Technology International, New Jersey). The scan rate was 1 nm/s with excitation at 395 nm and emission range from 420 to 550 nm. After measuring the background protein fluorescence, 7.5 *µ*L of ANS stock solution were added in the cuvette and mixed so that the final ANS concentration was 250 *µ*M. A control ANS spectrum in the absence of protein was also recorded to allow the calculation of ANS fluorescence enhancement in the presence of apoE3.

### DMPC remodeling kinetics

Dimyristoyl-L-α-phosphatidylcholine (DMPC) remodeling assays were performed as described previously [60], [61] with the following modifications: DMPC concentration was 97 µg/ml and apoE3 concentration was 43 µg/ml. The reaction was performed in DPBS with 3.5% KBr (w/v), 0.1 mM DTT, 0.1 M guanidine hydrochloride (GndHCl), 0.1 mM EDTA. Reaction kinetics were followed by the change in absorbance at 325 nm using a Perkin-Elmer (USA) Lambda 35 UV/VIS spectrophotometer. The cuvette (1 cm pathlength) was thermostated at 24±0.1°C using the Perkin-Elmer PCB 1500 Peltier temperature controller. The contents were mixed by repeated pipetting for 3 sec every 2 min. Experimental data were fitted to a two-phase exponential decay model using Graphpad Prism™.

### Dynamic Light Scattering Analysis

Dynamic light scattering (DLS) experiments were recorded on a Zetasizer nano series instrument (Malvern Instruments Ltd, UK) at 25°C. ApoE3 samples were at 0.1 mg/ml in DPBS.

### Statistical analysis

Equation fitting was performed by Graphpad Prism™. To test the reproducibility of the results we applied a paired student t-test to experimental data collected from separately refolded protein batches. Each experimental set was comprised by both the wild-type (WT) protein and all three variants. For thermal and chemical denaturation measurements as well as ANS binding, statistical analysis was performed using experimental data collected on separate days. For DMPC remodeling measurements all experiments were performed on the same day to minimize variability from the preparation of the DMPC vesicles.

## Results

### Protein expression and purification

All four proteins (wild-type apoE3 and variants R145C, K146E and R136S) were expressed by HTB13 cells after infection with recombinant adenovirus carrying the apoE3 gene with the desired mutation and purified to homogeneity by a lipidation/delipidation protocol as described in the materials and methods section. The location of the 3 mutations on the crystallographic structure of the N-terminal fragment of apoE3 is shown in Figure 1. SDS-PAGE analysis indicated that all four recombinant proteins migrated with identical profiles and were more than 98% pure (Figure 1). The presence of a higher MW band corresponds to sialylated forms of apoE3 as described previously [62], [63] and was confirmed by deglycosylating the protein using Clostridium perfringens Neuraminidase (not shown).

### Secondary structure of apoE3 variants

**Figure 2:**
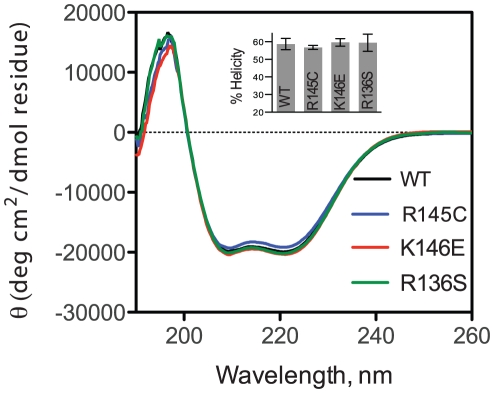
Circular dichroism spectra of wild-type apoE3 and variants (wild-type in black, R145C in blue, K146E in red and R136S in green). Spectra shown are the average of three separate measurements on different days with different protein batches. *Inset:* Helical content of WT apoE3 and variants calculated using the molar ellipticity at 222 nm as described in the materials and methods section. Error bars represent standard deviation based on three independent measurements.

Since all three mutations are located in the helical region of apoE3 (Figure 1A) we first investigated whether they affect the secondary structure of that region. We measured the circular dichroism signal of each apoE3 variant in the far UV region and compared it to the signal of the wild-type apoE3 protein. The overall shape of the CD spectrum in the region 190–260 nm was essentially identical between the variants and wild-type apoE3 indicating that the mutations did not affect the secondary structure of the protein (Figure 2). All three variants were estimated to carry similar amounts of helical content (Table 1 and Figure 2, inset). Although small differences were evident, these changes were not found to be statistically significant over the experimental variation between different protein preparations measured on different days. Overall, we conclude that the mutations R145C, K146E and R136S do not significantly affect the secondary structure of apoE3.

**Table 1:** Secondary structure content for apoE3 and variants calculated from Circular Dichroism spectroscopy experiments.

apoE3	% α-helix_222 nm_ 1	CDSSTR 2				
		% Helix	% Strand	% Turns	% Unordered	NRMSD^3^
WT	58.7±3.2	62	15	10	13	0.004
R145C	56.8±1.2	59	16	11	14	0.005
K146E	59.7±2.2	61	16	10	12	0.004
R136S	59.4±4.9	60	16	11	14	0.004

1 Values are means ± SD from three to four experiments.

2 prediction is averaged over sets 4,7 and SP175.

3 parameter refers to quality of fit of modeled spectra to actual spectra, see reference [57].

### Thermal denaturation measurements

**Figure 3:**
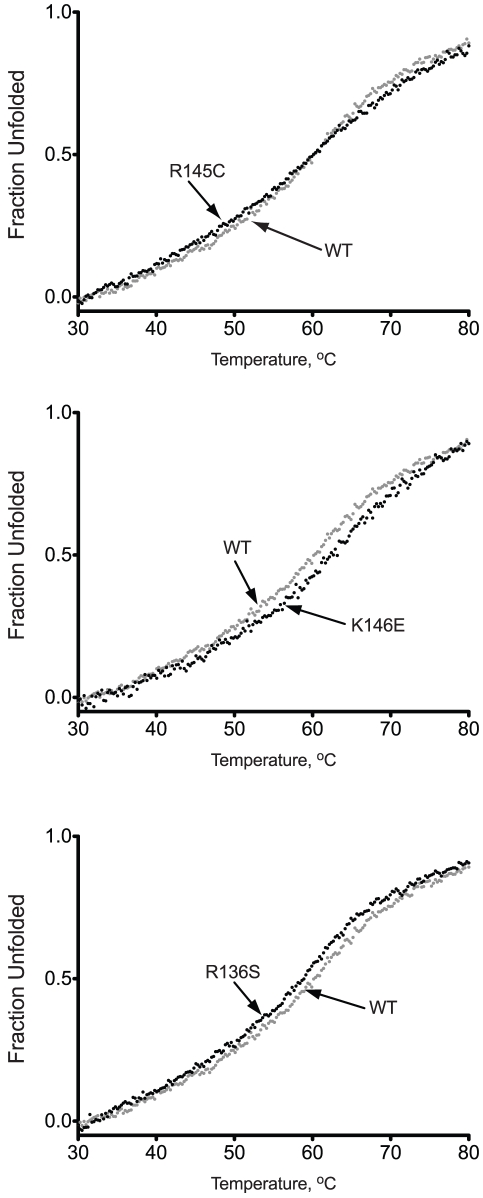
Thermal denaturation profiles of wild-type apoE3 and apoE3 variants. Each apoE3 variant (black dots) is presented in comparison with the wild-type protein (gray dots).

To test whether the apoE3 variants have altered thermodynamic stability, we followed the molecular ellipticity at 222 nm while the protein was gradually unfolded by increasing the temperature of the sample from 20 to 80°C. All three variants as well as wild-type apoE3 underwent a single, moderately cooperative transition to an unfolded state that contained significantly less helical content (Figure 3). The data were fit to a Boltzmann sigmoidal equation describing a transition between two states. Although the thermal denaturation of apoE3 is not a fully reversible process due to protein aggregation, such analysis has been used before as a method of comparing changes between apoE3 variants, primarily because the irreversible changes are relatively slow compared to the heat unfolding [64]. We therefore calculated the thermal midpoint of the transition, T_m_, the apparent cooperativity coefficient n, and the apparent enthalpy change of the transition ΔH (Table 2). ApoE3-K146E was found to undergo thermal denaturation at about 2°C over the wild-type protein whereas apoE3-R136S had a T_m_ at about 2°C lower compared to the wild-type protein. Although these changes are relatively small, they were found to be statistically significant (p<0.005) and suggest subtle changes in the distribution of conformations between variants. ApoE3-R145C presented the most prominent changes in thermal denaturation profile. Although the T_m_ of apoE3-R145C was identical to that of the wild-type protein, the shape of the transition was less steep as evident from the change in the cooperativity coefficient n (Figure 3 and Table 2). As a result, the apparent ΔH of the transition for apoE3-R145C was decreased by almost 7 kcal/mol compared to the wild-type protein and the other two variants indicating that this single amino acid change can have significant impact on the thermal stability of apoE3 (Table 2).

**Table 2:** Parameters calculated for the thermal unfolding of apoE3 and variants.

apoE3	Tm (°C)	n^1^	apparent ΔΗ (kcal/mol)^1^
WT	53.6±0.2	7.7±0.2	27.9±0.1
R145C	53.4±0.3	6.9±0.4^***^	21.3±1.4^****^
K146E	**55.7±0.1** *	7.9±0.2	27.3±1.1
R136S	51.9±0.4^**^	7.6±0.6	27.4±0.8

Values are means ± SD from three to four experiments.

*p = 0.002, ** p = 0.004, *** p = 0.015, **** p = 0.007.

1parameters calculated assuming a reversible two-state model.

**Figure 4:**
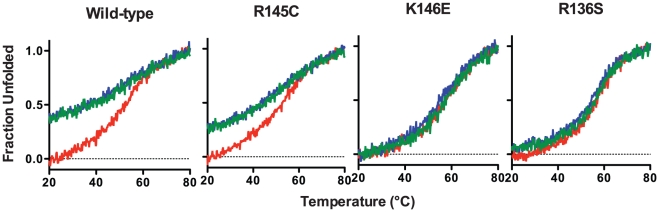
Reversibility of the thermal denaturation of WT apoE3 and variants. ApoE3 was thermally unfolded while following the CD signal at 222 nm (red trace). After reaching 80°C, the protein was gradually refolded by cooling the sample chamber down to 20°C (blue trace). The protein sample was then thermally unfolded again (green trace).

Consecutive heating and cooling of apoE3 samples was utilized to gauge the reversibility of the thermal unfolding. It has been demonstrated before that apoE3 thermal unfolding is only partially reversible due to the slow aggregation of the protein [64]. To investigate whether the variants share this property, we conducted consecutive heating, cooling and re-heating on each apoE3 sample (Figure 4). Wild-type apoE3 showed a mostly non-reversible transition with a close to 60% signal recovery upon cooling. The second unfolding transition however, did demonstrate similar sigmoidal shape and T_m_ to the first indicating that part of the protein ensemble was able to undergo a reversible transition. ApoE3 variant R145C demonstrated a similar pattern with minor differences in the amount of CD signal recovery upon refolding. Surprisingly, apoE3 variants R136S and K146E demonstrated a predominantly reversible transition, recovering about 95-99% of the signal upon cooling. This finding suggests that these variants may have different competing kinetics between refolding and thermally induced aggregation, indicative of changes in the conformational plasticity of the protein.

### Chemical denaturation measurements

**Figure 5:**
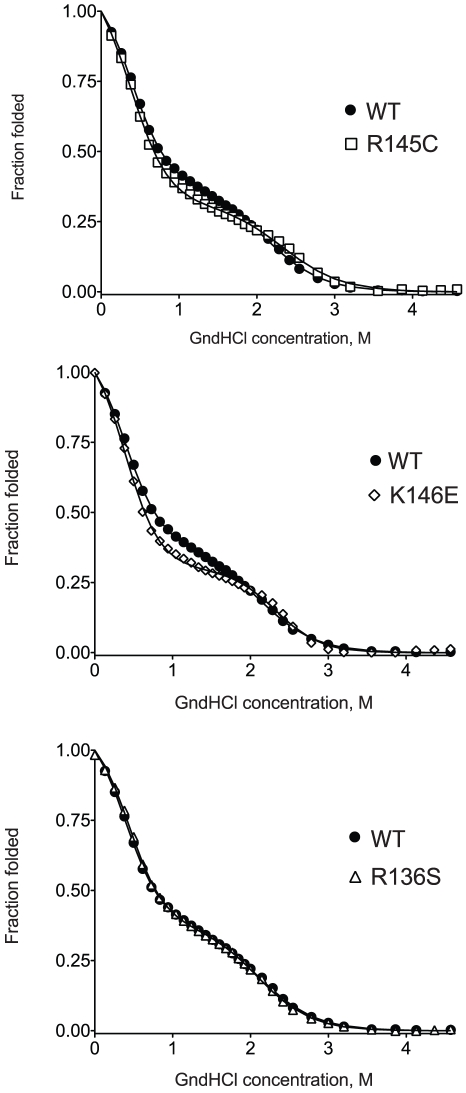
Chemical denaturation profiles of wild-type apoE3 and apoE3 variants. Each variant is presented in comparison with the wild-type protein (black circles). Solid lines represent non-linear regression fits to a three-state unfolding model as described in the materials and methods section.

To further investigate possible changes in the thermodynamic stability of apoE3 due to the presence of mutations R145C, K146E and R136S we studied the chemical denaturation profile of the apoE3 variants (Figure 5). The tryptophan fluorescence signal of each protein variant was followed as a function of increasing concentration of the chaotrope guanidine hydrochloride. All three variants exhibited similar shape curves as wild-type apoE3 with a characteristic shallow plateau near the midpoint of the denaturation, indicative of a transition intermediate state as described before [43]. To analyze the thermodynamics of these transitions we fitted the experimental data to a 3-state denaturation model that allowed us to calculate the apparent change of the free Gibbs energy for each transition (see Materials and Methods)[59]. The results of the fit are shown in Table 3. ApoE3-R145C and apoE3-R136S had a ΔG of 4.6±0.2 and 4.8±0.2 kcal/mol respectively, values that did not statistically differ from the value calculated for the wild-type protein (4.4±0.2 kcal/mol). ApoE3-K146E however had a ΔG of 6.0±0.3 kcal/mol, a value that was about 1.6 kcal/mol higher than the wild-type protein (p = 0.08) indicating stabilization of this variant compared to the wild-type protein. This stabilization was found to be primarily due to the stabilization of the second unfolding transition of apoE3 variant (Table 3) that corresponds to the unfolding of the N-terminal domain where the K146E mutation is located [13], [44], [65].

**Table 3:** Parameters calculated from chemical denaturation and ANS binding experiments for wild-type and apoE3 variants.

apoE3	app. ΔG_1_ (kcal/mol)	app .ΔG_2_ (kcal/mol)	app. ΔG_total_ (kcal/mol)	m_ni_ 1(kcal mol^-1^ M^-1^)	m_iu_ 1(kcal mol^-1^ M^-1^)	x_ni_ 2 (M)	x_iu_ 2 (M)	ANS binding (fold increase)
WT	1.0±0.2	3.4±0.1	4.4±0.2	2.6±0.2	1.6±0.1	0.39±0.02	2.09±0.03	3.6±0.5
R145C	0.9±0.2	3.6±0.2	4.6±0.2	2.5±0.2	1.6±0.2	0.36±0.08	**2.33±0.04** *4	**4.2±0.4** *6
K146E	1.0±0.2	**4.9±0.3** *1	**6.0±0.3** *2	2.7±0.2	**2.1±0.3** *3	0.39±0.03	**2.32±0.03** *5	3.9±0.4
R136S	1.2±0.2	3.7±0.1	4.8±0.2	2.7±0.2	1.7±0.1	0.44±0.03	2.09±0.03	3.9±0.5

Values are means ± SD from three to four experiments.

*^1^p = 0.07,

*^2^p = 0.08,

*^3^p = 0.08,

*^4^p = 0.024,

*^5^p = 0.06,

*^6^p = 0.03

1 x_ni_ and x_iu_ are the mid-transition points for the transition from the native to the intermediate state and from the intermediate to the unfolded state respectively.

2 m_ni_ and m_iu_ correspond to the slope of the mid-transition points from the native to the intermediate state and from the intermediate to the unfolded state respectively.

### ANS binding measurements

**Figure 6:**
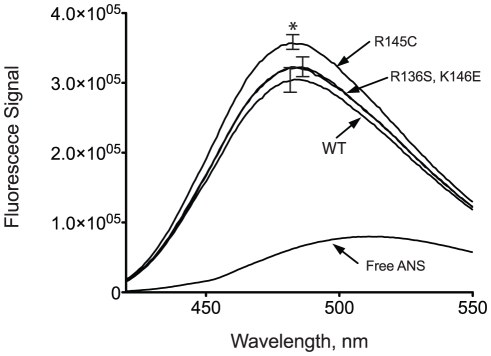
ANS fluorescence spectra in the presence or absence of wild-type apoE3 and variants. Spectra are averages of three independent experiments. Error bars indicate standard deviation based on three independent measurements. *p = 0.03.

Binding of ANS onto apoE has been previously used to probe the hydrophobic binding properties of the molecule and as a measure of possible conformational rearrangements [43]. To examine whether the mutations under study here affect the solvent exposure of hydrophobic regions of the protein we measured ANS fluorescence in the presence of wild-type apoE3 and its variant forms (Figure 6). Binding of ANS to apoE3 resulted in a marked increase of its fluorescence signal accompanied by a blue-shift consistent with ANS binding to hydrophobic sites on the protein. ApoE3 variants R136S and K146E did not show any statistically significant change in ANS fluorescence compared to wild-type apoE3 (Figure 6 and Table 3). However, apoE3 variant R145C did demonstrate enhanced ANS fluorescence over wild-type apoE3. These measurements are consistent with the nature of the mutated amino acid, since we observed increased ANS fluorescence only for the apoE3 variant in which a hydrophilic residue (Arg) is substituted for a more hydrophobic residue (Cys). It is notable however, that a single amino acid substitution can lead to a sufficiently enhanced, solvent-accessible, hydrophobic surface that can affect ANS binding.

### DMPC remodeling kinetics

**Figure 7:**
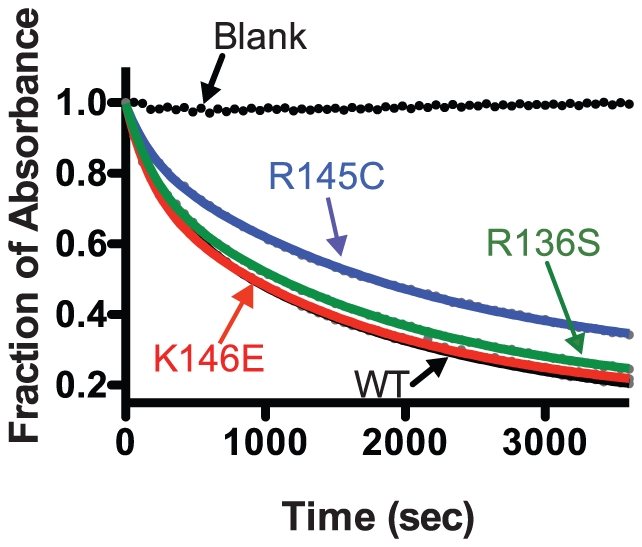
Time course of remodeling of multilamellar DMPC vesicles by wild-type apoE3 and apoE3 variants. Absorbance at 325 nm was followed for 1 hr after addition of apoE3 to DMPC vesicles as described in the materials and methods section. Experimental data (closed circles) were fit to a two-phase exponential decay model (solid lines).

ApoE interacts with lipids by a complex mechanism that includes significant conformational changes including the partial unfolding of its N-terminal domain [47], [48], [60], [61], [66], [67]. To test whether the mutations studied here can affect this process we measured the kinetics of DMPC vesicles remodeling in the presence of the apoE3 variants (Figure 7 and Table 4). All three variants were efficient in clearing DMPC vesicles with relatively slow kinetics, similarly to the WT apoE3. The data were fit to a bi-phasic exponential decay equation as described in Materials and Methods, allowing the accurate determination of the slow and fast component half-lives (Table 4). This analysis indicated small, but statistically significant, differences in the slow component of the decay for variants R145C and R136S. These changes may be related to the kinetics of the N-terminal four-helix bundle opening upon interactions with phospholipids.

**Table 4:** Parameters calculated from DMPC remodeling experiments for apoE3 variants.

apoE3	t_1/2 fast_(sec)	t_1/2 slow_(sec)	% plateau
WT	176±92	1039±143	17±13
R145C	133±41	**1416±69** *	24±3
K146E	145±62	1247±303	14±2
R136S	165±85	1378±178**	15±4

Data were fit to a two-state exponential decay model. Half-lives for each component of the decay are shown. Values are means ± SD from three experiments.

*p = 0.012, **p = 0.004.

### Dynamic Light Scattering Measurements

**Figure 8:**
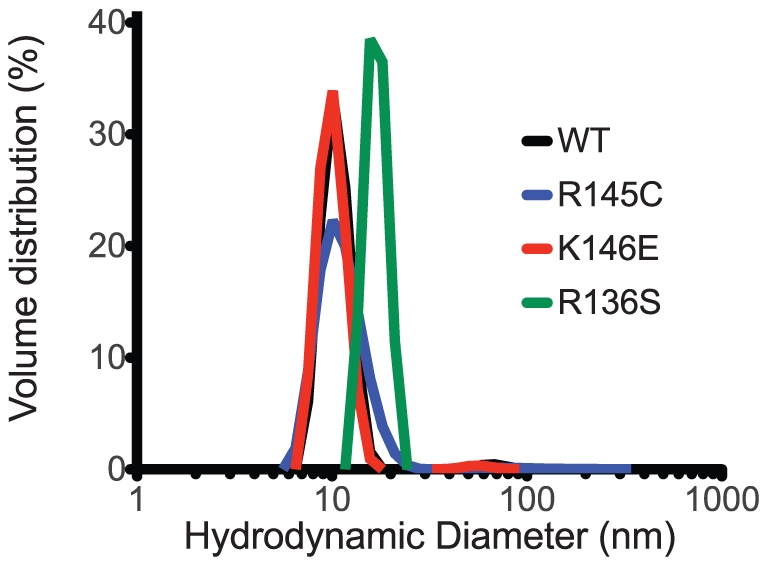
Volume-normalized distribution of particle hydrodynamic diameters in 0.1 mg/ml samples of WT apoE3 and variants. Peak analysis parameters are shown in Table 5.

ApoE has been shown to exist in oligomeric states in solution, ranging from dimers to octamers, but predominantly tetramers, mediated by interactions of its C-terminal domains [14], [49], [64]. To test the oligomerization state of the variants we used Dynamic Light Scattering to determine each variant's hydrodynamic diameter in solution, at the concentration range typically used for biophysical analysis (0.1 mg/ml). The particle size distribution, normalized by intensity and volume occupancy in the ensemble is shown in Figure 8. In all cases >99% of the scattering mass was found to originate from species in the 10–20 nm diameter range, with <1% of the mass corresponding to higher-order aggregates. Both the WT apoE3 and variant K146E demonstrated a similar distribution with a predominant particle diameter of 10.4±1.7 and 10.1±1.7 nm respectively (Figure 8 and Table 5). The R145C variant had a similar hydrodynamic diameter but with a slightly larger distribution possibly indicating a tendency towards aggregation. Interestingly, the R136S variant demonstrated a significantly higher hydrodynamic diameter in solution (16.9±2.1 nm) suggesting that it exists in a higher-order oligomerization state compared to the wild-type protein.

**Table 5:** Parameters calculated from DLS experiments for apoE3 variants (Figure 8).

apoE3	Diameter (I)**Peak 1	Diameter (I)**Peak 2	Diameter (V)**
WT	10.9±1.4	68 ±7	10.4±1.7
R145C	13.7±3.6	137±67	11.3±3.1
K146E	10.6±1.4	58±6	10.1±1.7
R136S	17.1±1.2	n/d*	16.9±2.1

Calculated particle diameters are given in nm. Errors indicate peak width. Peak 2 corresponds to ∼1% of the total mass of the protein sample and indicates higher order aggregates. Units are in nm. Values are means ± SD from three experiments.

*non-detected.

**I =  diameter calculated based on the distribution of intensities of scattering, V =  diameter calculated after normalization of the scattering distribution based on particle volume occupancy.

## Discussion

**Figure 9:**
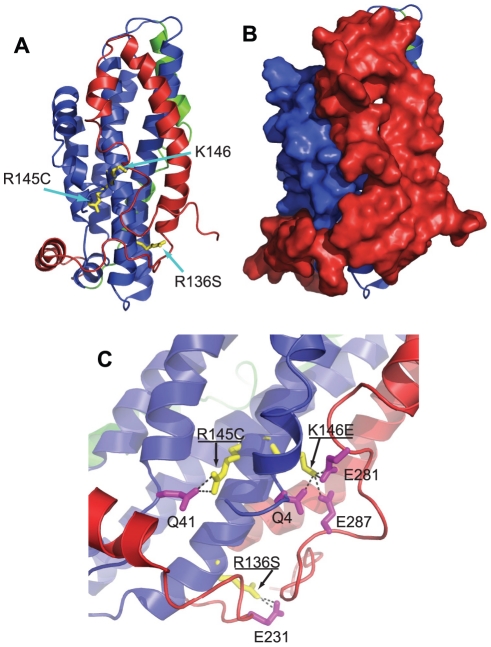
*Panel A,* Location of amino-acid substitutions in the apoE3 variants under study in the recently determined structure of a monomeric variant of apoE3 (pdb code 2L7B). Protein is shown in cartoon representation with the N-terminal domain colored in blue, the C-terminal domain colored in red and the hinge-domain colored in green (see reference [68]). *Panel B,* same as in panel A, but with the C-terminal and N-terminal domain shown in surface representation to demonstrate that positions 136, 145 and 146 are not solvent exposed in this structure. *Panel C,* interactions between amino acids R136, R145 and K146 and the C-terminal or N-terminal domain of the protein. All three amino acids are stabilized by specific interactions with either charged residues of the C-terminal domain (for K146 or R136) or with hydrogen bonding within the N-terminal domain [68]. Images were prepared using PyMol 1.3 (www.pymol.org).

The postulated multiple conformational states of apoE3 make it challenging to unequivocally provide a structural explanation for the observed changes in the protein's biophysical properties. Up until recently, direct structural information existed only for the N-terminal segment of apoE3 that has the topology of a four-helix bundle (pdb code, 1LPE, Figure 1). In this structure, the amino acid positions studied here are all largely exposed to the solvent and are thought to mediate interactions with the LDL receptor. During the authoring of this manuscript however, a new structure of a monomeric mutant of apoE3 was published, a structure that provides an invaluable snapshot of the apoE3 conformational ensemble in solution [68]. In this structure (pdb code: 2L7B) the N-terminal segment of the protein is also folded in a four-helix bundle (Figure 9A) with the locations of amino acids R136, R145 and K146 in similar configurations as in structure 1LPE. The C-terminal domain is highly helical and folded around the N-terminal domain in such a configuration to completely protect the LDL-receptor binding region 136-150 from the solvent (Figure 9B). As a result of this interaction, Chen et al proposed that in order for apoE3 to interact with the LDL-receptor, it has to partially unfold to expose this site[68]. All three amino acids under study here participate in specific inter-domain interactions that stabilize their charged/hydrophilic nature allowing their sequestration from the solvent (Figure 9C). These interactions are expected to be abrogated in the variants under study here, essentially perturbing important domain interactions in apoE3. Changes in inter-domain interactions can influence folding and unfolding transitions leading to the observed effects in the biophysical behavior of the apoE3 variants. Inter-domain interactions within apoE have been hypothesized to underlie key conformational changes related to apoE function [44], [45]. Although it is difficult to unequivocally explain these phenomena without knowing the protein's unfolding and folding pathways, some interesting correlations can be derived. For example, variant R136S is found to be prone to higher-order oligomerization and it participates in interactions with E231 a residue suggested to participate in the oligomerization of apoE3 based on recent studies [69], [70]. Furthermore, variant R145C was found to expose additional hydrophobic surfaces to the solvent and it is expected to destabilize the interaction between amphipathic helices N1 and 4 possibly leading to partial exposure of the intra-helix core[68]. Finally, variants K146E and R136S are expected to perturb interactions between the N- and C-terminal domains and are found to grossly affect the unfolding reversibility of apoE3, a transition that would be expected to be dominated by the ability of these two domains to interact reversibly.

Single amino acid substitutions in the N-terminal moiety of apoE have been shown before to affect the proteins biophysical behavior, function and role in disease pathogenesis. Specifically, functional differences between apoE4 and apoE3 have been attributed to altered inter-domain interactions brought about by the polymorphism at position 112 of the N-terminal domain. This polymorphic variation brings about alterations in the thermodynamic properties of the molecule that may underlie functional changes linked with disease predisposition [61], [65], [71], [72], [73], [74]. It is conceivable that the three mutations studied here influence the thermodynamic stability and conformation of full-length apoE3 in a similar manner. Similarly, differences in function and disease predisposition are established between apoE3 and apoE2 although these two variants differ only at a single amino acid at position 158 [65], [72], [74]. In this context, the finding that rare pathogenic mutations in the N-terminal domain of apoE3 affect thermodynamic stability parameters of the full-length molecule is consistent with the well established effects of common apoE polymorphisms that are under intense scrutiny due to their role in the pathogenesis of cardiovascular and Alzheimer's disease [45], [74].

The precise molecular mechanism that links specific apoE mutations with dominant forms of type III HLP remains unclear. In many cases the studied molecular properties of apoE variants associated with the disease do not correlate well with disease penetrance or severity indicating that other factors may also contribute to the pathogenesis of the disease. For example, apoE2, which is associated with a recessive form of type III HLP, binds to the LDL receptor 50-fold weaker than apoE3 [22], [75]. In contrast, all apoE3 variants studied here demonstrate stronger LDL receptor binding compared to wild-type apoE2 [28], [30], [34], [35]. Still, these variants not only maintain their ability to predispose to type III HLP, but also do so in a dominant fashion, unlike apoE2 [22], [26], [30], [31], [33], [34], [35]. These observations suggest that these variants must carry additional properties that contribute to their pathogenic potential.

In this study we hypothesized that type III HLP predisposing mutations may lead to alterations in the structure and thermodynamic stability of apoE3 since such alterations have previously been linked with the pathogenic potential of the protein. We further hypothesized that the common pathogenic potential of these variants may be derived by similar thermonynamic or structural consequences. Indeed, our findings point to specific changes in the thermodynamic properties of the variants. These changes however are distinct between the three variants suggesting that they do not constitute a single unifying mechanism leading to protein dysfunction and that further functional analysis will be necessary to clarify their role in apoE3 function. It is possible however, given the importance of structural plasticity of apoE3 to its function, that the molecule's thermodynamic stability is optimized to provide sufficient stability in the lipid-free form but at the same time sufficient lability to allow unfolding transitions upon lipid interactions. In this context any deviation from this optimized state, regardless if they are destabilizing or stabilizing may signify altered dynamics between different apoE3 conformations in solution that are detrimental to apoE3 function.

In summary, in this study we analyzed the biophysical properties of three apoE3 variants that predispose to the development of type III HLP. We found statistically significant differences in several parameters examined indicating that these single-amino acid variations can affect the stability and conformational distribution of apoE3 in solution. We were unable however to discern a unifying pattern between the observed thermodynamic alterations in each variant that could be used to develop a single thermodynamics-based hypothesis regarding their pathogenic potential. Although it is possible that the thermodynamic alterations in each variant lead to common dysfunctions in the structural plasticity of apoE3, the relatively small magnitude of the perturbations makes it less likely that they constitute a primary factor. Regardless, these alterations in the biophysical properties of these variants should be taken into account in further functional analysis aiming to clarify the link between apoE3 and the development of type III HLP.
